# Trustworthy Localization in IoT Networks: A Survey of Localization Techniques, Threats, and Mitigation

**DOI:** 10.3390/s24072214

**Published:** 2024-03-29

**Authors:** Giovanni Pettorru, Virginia Pilloni, Marco Martalò

**Affiliations:** 1 Department of Electrical and Electronic Engineering, University of Cagliari, 09123 Cagliari, Italy; giovanni.pettorru@unica.it (G.P.); virginia.pilloni@unica.it (V.P.); 2 Research Unit of Cagliari, National Inter-University Consortium for Telecommunications (CNIT), 09123 Cagliari, Italy

**Keywords:** internet of things (IoT), wireless sensor network (WSN), localization, security, trustworthiness, threats models

## Abstract

The Internet of Things (IoT) has revolutionized the world, connecting billions of devices that offer assistance in various aspects of users’ daily lives. Context-aware IoT applications exploit real-time environmental, user-specific, or situational data to dynamically adapt to users’ needs, offering tailored experiences. In particular, Location-Based Services (LBS) exploit geographical information to adapt to environmental settings or provide recommendations based on users’ and nodes’ positions, thus delivering efficient and personalized services. To this end, there is growing interest in developing IoT localization systems within the scientific community. In addition, due to the sensitivity and privacy inherent to precise location information, LBS introduce new security challenges. To ensure a more secure and trustworthy system, researchers are studying how to prevent vulnerabilities and mitigate risks from the early design stages of LBS-empowered IoT applications. The goal of this study is to carry out an in-depth examination of localization techniques for IoT, with an emphasis on both the signal-processing design and security aspects. The investigation focuses primarily on active radio localization techniques, classifying them into range-based and range-free algorithms, while also exploring hybrid approaches. Next, security considerations are explored in depth, examining the main attacks for each localization technique and linking them to the most interesting solutions proposed in the literature. By highlighting advances, analyzing challenges, and providing solutions, the survey aims to guide researchers in navigating the complex IoT localization landscape.

## 1. Introduction

The Internet of Things (IoT) has rapidly revolutionized humans’ interactions with the environment and reshaped their daily routines. This technology is applicable across a wide spectrum of sectors, ranging from smart homes and healthcare to industrial automation and transportation, taking a central role in each. To grasp the profound impact of IoT on our lives, consider that there are currently an estimated 15 billion connected devices worldwide, and this number is poised to increase in the coming years, steadily [1]. The increasing promise of the IoT for specific applications like home automation, smart farming, and industry 4.0 can be largely attributed to the rising prevalence and advancements in Machine-to-Machine (M2M) communication [2]. This represents a departure from the conventional Machine-to-Human (M2H) interactions that we have been accustomed to through the traditional Internet.

In this evolving landscape, where devices increasingly require less human interaction, the significance of context awareness becomes paramount. The term context awareness, which originated more than two decades ago, can be defined in the context of IoT as the ability of devices to collect and use data about their surroundings, enabling them to make more informed and context-relevant decisions [3]. The data collected, referred to as contextual information, encompass a wide range of categories, including location, timestamp, user behaviors, proximity to other devices, battery level, and various other factors. One of the most interesting contextual information in the IoT landscape is the location of objects in the application scenario, which gives rise to a distinct category of services known as Location-Based Services (LBS) [4]. Focusing on the IoT domain, one can find a variety of LBS cases in the literature. These include the provision of navigation guidance for warehouse robots [5], the ever-expanding realm of location-based marketing [6], and services to safeguard the well-being of elderly people through precise tracking of their activities and movements in the comfort of their homes [7]. These are just a few examples that give an idea of the many ways in which LBS can significantly affect humans’ daily lives. As the use of IoT technologies has grown, so have the security issues associated with it [8]. Nowadays, a secure-by-design approach is an absolute imperative when designing IoT systems, particularly those as sensitive and vulnerable as location-related ones.

Building on the premises established in this introduction, this study aims to provide a comprehensive review of the literature on IoT localization techniques, threat models, and solutions, differentiating itself from existing surveys. The key contributions of this survey are as follows:

- C1: an innovative methodology that thoroughly reviews the existing literature on IoT localization, integrating techniques, and security considerations.
- C2: a classification of active radio-based localization techniques into range-based and range-free, with emphasis on understanding and comparing the different approaches while discussing their applicability to different use cases.
- C3: a mapping between each localization method and the corresponding threat models and proposed mitigation solutions documented in the literature.
- C4: guidelines to help researchers identify key references in the literature, serving as a valuable resource to facilitate the progress of their work in this specialized field.

The rest of this survey is structured as follows. In Section 2, we illustrate the methodological approach we have followed in our review, as well as the reference scenario, and briefly summarize the state of the art in terms of surveys touching different aspects of IoT localization, including techniques, threat models, and solutions (C1). Section 3 provides a broad review of the state of the art of localization techniques used in IoT environments (C2). In Section 4, our attention turns to exploring the threat models, detection, and mitigation approach for localization systems that have been documented in the existing literature (C3). To conclude, Section 5 and Section 6 offer a summary of the survey results, providing guidelines to enhance the readability of the paper, discussing the state of the art gathered from the survey, and making concluding remarks (C4).

A list of acronyms that are used in the manuscript is given in Table 1 to facilitate readability.

## 2. Background

### 2.1. Methodological Approach

Our literature search work for writing this survey follows a well-defined methodology. We mainly used major search databases, including *MDPI*, *IEEE Xplore*, *Elsevier*, and *Springer* for the systematic selection of the most relevant articles. The selection process was guided by specific keywords, namely, *localization*, *IoT*, *secure*, *reliable*, *attack*, and *malicious*. To maintain the highest level of accuracy and topicality in our survey, we chose our search criteria precisely, narrowing our focus to works published since 2018. In addition, regarding articles with more than 1 year since their publication, we prioritized those that obtained citations, thus incorporating their literary impact into our analysis.

The survey comprises two blocks. The first extensively covers IoT localization techniques, offering a comprehensive domain overview. In the subsequent block, we delve deeply into the primary categories of attacks on localization systems, categorizing them based on the malicious user’s intent and presenting identification methods for each. This segmentation enhances our understanding of the diverse threat landscape and its varied impacts on the localization process.

### 2.2. Review of Related Survey Works

In the literature, numerous research studies have individually addressed different aspects of IoT localization, including techniques, threat models, and solutions. In Table 2, we present a comparative analysis of these works, highlighting their main areas of interest. The following is a brief summary of these main areas, i.e., IoT localization techniques, threat models, and their corresponding solutions.

From the perspective of IoT localization techniques, several surveys can be found in the literature, which underscore the growing interest of researchers in this particular area. The authors of [9] offer a comprehensive analysis of localization techniques, alongside the development of a hierarchical taxonomy. This study classifies the localization approaches into two distinct categories within the context of IoT scenarios: Self-Determining Method and Training-Dependent Method. In [10,11,12], the authors propose a comprehensive survey, simultaneously conducting evaluations using metrics such as energy efficiency, availability, cost, reception range, latency, scalability, and accuracy. The approach in [13] aligns with previous methods, with an added analysis addressing error sources and their mitigation. The work presented in [14] focuses on different outdoor and indoor environments and various contexts, including Wireless Sensor Networks (WSNs), IoT, cognitive radio networks, and 5G networks. In contrast, the works proposed in [15,16] focus their attention on specific IoT scenarios, those outdoors, evaluating the accuracy and robustness of the algorithms in harsh environments characterized by obstacles such as buildings. The studies presented in [17,18] focus their survey on Machine Learning (ML)-based fingerprinting localization approaches, providing insight into a previously underexplored branch. Even the work discussed in [19] delves into approaches that have received limited attention in the literature, offering a survey that focuses on visible light-based localization and assesses its potential. Recent comprehensive surveys on these aspects are [20,21], notable for their up-to-date coverage of this evolving field.

**Table 2:** Summary of surveys dealing with IoT localization techniques, threats, and solutions.

Areas of Interest	Year	Reference	Distinctive Characteristics
IoT localization techniques	2018	[9]	Compare and categorize existing works within an IoT infrastructure framework, and offer a comprehensive taxonomy.
	2019	[10]	Evaluate different proposed systems through key IoT requirements
		[11]	Investigate the impact of localization in the modern IoT and the main challenges
		[14]	Focus on MDS-based localization techniques for several scenarios
	2020	[13]	Overview of error sources and mitigation, performance evaluation, and an analysis of the applications, opportunities, and challenges.
		[17]	Focus on ML and intelligent algorithms for Fingerprint-Based techniques
		[19]	Overview of promising techniques based on visible light
		[15]	RF-based localization in Smart Cities scenarios
	2021	[16]	GNSS-free outdoor localization techniques
		[12]	Comparative analysis based on different performance parameters
		[18]	ML-based Wi-Fi RSS fingerprinting schemes and investigation of training datasets in the literature
	2022	[20]	In-depth analysis of LBSs, latest applications, and major vendor profiles
		[21]	Focus on the strengths and weaknesses inherent in each localization technology and technique
Threats models and solutions	2017	[22]	Security and privacy for LBS from a technical and legal perspective
	2020	[23]	Advances in location privacy protection technology in the context of SIoV
	2022	[24]	Privacy attacks in location and corresponding solutions, with a focus on VANETs
Joint analysis	Our survey aims to cover this gap in the literature by presenting a joint analysis of IoT localization techniques, threats models, and solutions		

In the recent literature, there is a scarcity of comprehensive studies dedicated to addressing security issues in the context of IoT localization. Among the most interesting contributions, we find [22], where several issues related to security and privacy in IoT-based location systems are analyzed, with a focus on both the technical and legal perspectives. Continuing our analysis in the specific intersection of IoT location and vehicular networks, the survey described in [23] systematically examines recent advances in location privacy protection technology in the context of the Social Internet of Vehicles. In addition, the authors introduce and evaluate the performance of three distinct types of user data privacy protection technologies. With the same focus on vehicular ad hoc networks, the authors of [24] offer a comprehensive review of location privacy attacks and propose solutions to mitigate the problems arising from such attacks in these particular IoT applications.

In contrast to the studies described in this section, our study seeks to make a more substantial contribution by providing an overview aimed at paving the way for researchers in this field. Our survey includes the following key elements in a single document:

- A comprehensive review of the main IoT localization techniques and algorithms found in the literature;
- An in-depth investigation of hybrid solutions, an emerging approach that has not been comprehensively explored in existing literature surveys;
- An analysis of associated security threats, along with potential solutions proposed in the literature for each category.

### 2.3. Reference Scenario

**Figure 1:**
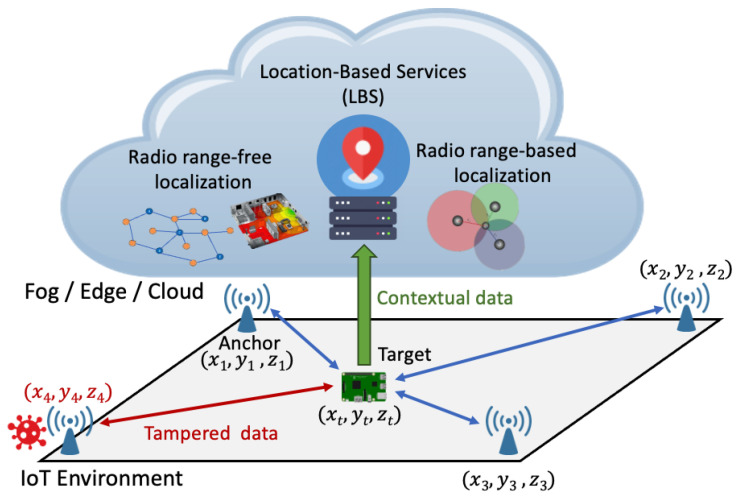
Reference scenario for the proposed analysis [20,25].

The reference scenario for our analysis is visually represented in Figure 1 [20,25]. This scenario involves the deployment of $N_{\mathrm{anchors}}$ wireless devices acting as anchors, each of which has known locations, and a target whose location is to be estimated. The *i*-th anchor deployed in the environment (*i* = 1, 2, …, $N_{\mathrm{anchors}}$) has coordinates $a_{i}=\left[x_{i},y_{i},z_{i}\right]$, whereas the target has coordinates $t=[x_{t},y_{t},z_{t}]$. To facilitate localization, anchors transmit packets to the target, which collects and analyzes them. Depending on whether the localization system is device-based, device-assisted, or network-based, the collected measurements are used for localization estimation directly by the target or are routed to the gateway toward more powerful devices for further processing. Consider that in an IoT environment, where connectivity such as Wi-Fi is already established, the existing infrastructure can be leveraged instead of introducing additional devices. In this context, Access Points (APs) and any connected device can effectively take on the role of anchors as shown in the reference scenario. Note that our survey focuses exclusively on radio-based techniques. Therefore, our investigation does not include methods that do not depend on radio information, such as those that rely on inertial sensors.

In our reference scenario, it is critical to recognize the potential risks posed by malicious users. This study, in particular, delves into vulnerabilities at the physical layer within the architecture, highlighting potential threats from malicious users capable of infiltrating the network and gaining control of one or more anchors. The primary goal of a malicious user could be twofold: first, to intentionally manipulate the positioning system by introducing erroneous data (e.g., false reference position), thus causing an incorrect estimate of the target’s location; second, to disrupt the entire location process by compromising multiple anchors or making them inoperable (e.g., jamming attacks). The methods in which an attacker can interfere with these localization systems will be described comprehensively in detail in Section 4. The presence of a potential attacker who can compromise the security of a subset of $M<N_{\mathrm{anchors}}$ anchor nodes is assumed.

Even though attacks on the deeper parts of the infrastructure (e.g., fog, edge, and cloud layers) can have an impact on the localization process, this discussion falls beyond the scope of this manuscript. The focus of this survey is indeed on the local IoT network and the corresponding wireless communications among the nodes inside it.

## 3. IoT Localization Techniques

IoT localization techniques can be classified into two main groups: radio range-free and radio range-based methods [26]. Radio range-free techniques, on the other hand, do not rely on direct distance measurements. Instead, they exploit information such as network connectivity patterns, the number of hops between nodes, or the knowledge of the radio environment to approximate the position of the target [27]. Radio range-based localization involves measuring distances between anchor points and target devices; these distances are then used to estimate the target’s position using various approaches, including methods based on triangulation and multilateration [28]. The selection between these two categories depends largely on the specific needs and limitations of the IoT application. Radio range-based techniques offer higher accuracy but require additional hardware, involve higher energy consumption, and often involve complex implementation. In contrast, radio range-free techniques, which require no additional hardware, are energy efficient, and have a simpler configuration, generally offer lower accuracy [29].

Finally, there is a third category of hybrid localization approaches that aim to exploit the advantages and limit the drawbacks of different techniques and technologies to improve performance [30]. These methods aim to enhance the localization accuracy and robustness by *joining* different techniques, such as combining proximity with multilateration-based approaches. Alternatively, they leverage a single technique but achieve integration with multiple communication technologies through a *data fusion* approach, for instance, by combining Wi-Fi and Bluetooth data.

When focusing on nodes involved in the localization system, one more classification can be established: active nodes, which involve data transmission, and passive nodes, such as Reconfigurable Intelligent Surfaces (RISs) that rely on reception and reflection [31,32] to improve the efficiency of localization systems, especially in difficult environments such as shaded areas. Even though this second category of devices is worthy of investigation, this goes beyond the scope of this review. Therefore, in this review, we will focus primarily on systems that use exclusively active nodes.

**Figure 2:**
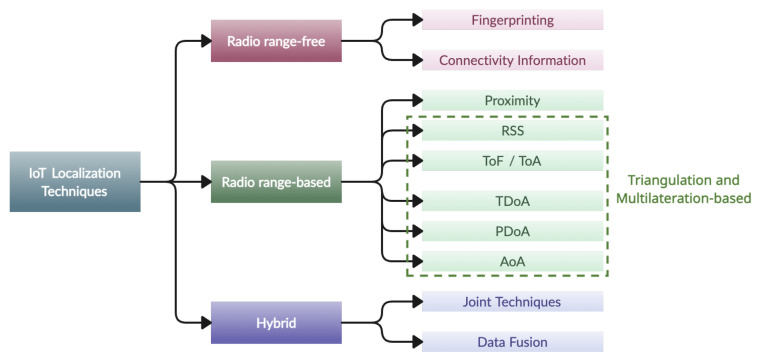
Taxonomy of IoT localization techniques.

In this section, we present an in-depth review of the literature, focusing on the main localization techniques used in the IoT environment. Figure 2 presents a comprehensive taxonomy. To facilitate navigation within the proposed categorization and provide information on the works discussed, a summary table will be presented at the end of the section.

### 3.1. Radio Range-Free

**Figure 3:**
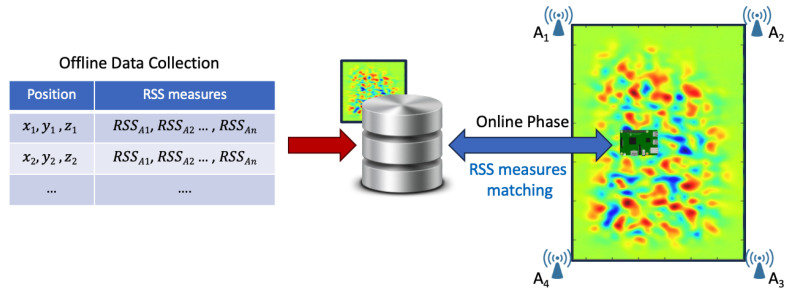
Fingerprinting-based localization.

*Fingerprinting*—This is the dominant radio range-free localization technique, as shown by the numerous existing works in the literature. This technique consists of two phases, illustrated in Figure 3. In the offline phase, Channel State Information (CSI) and/or RSS measurements are collected at various locations in the scenario to build a fingerprint database. In the online phase, the target moves within the scenario, acquiring RSS measurements that are compared with database entries to estimate its most likely location [33].

While this method offers the advantage of avoiding complex calculations for position estimation, its dependence on the offline phase makes it unsuitable for dynamic scenarios since significant changes in the environment would require it to be repeated. In [34], the authors introduce an innovative Wi-Fi fingerprinting-based approach for indoor scenarios, addressing the challenges of the offline phase through a dynamic radio map update system that eliminates the need for costly and time-consuming manual surveys.

This localization technique relies heavily on ML methods for accurate position estimation. Various models, including Random Forest, K-Nearest Neighbors (KNNs), Long Short-Term Memory (LSTM), and Convolutional Neural Networks (CNNs), are used for position estimation, each of which offers different advantages and capabilities as can be seen from the various works in the literature. The authors of [35] introduce a new application of the standard Particle Swarm Optimization (PSO) algorithm to improve indoor fingerprint-based localization. The study demonstrates a significant improvement in accuracy over Random Forest-based approaches. Wi-Fi returns to center stage in [36], presenting EdgeLoc, an indoor localization system designed to address hardware-induced RSS variations, multipath reflections, and computational constraints. Leveraging Capsule Neural Network models, a multistep data stream for RSS fingerprint processing, and an edge–IoT framework, EdgeLoc achieves real-time localization with high performance compared to standard approaches. Shifting focus, ref. [37] addresses precise localization in indoor scenarios with a heterogeneous infrastructure, including devices like Raspberry Pi and Arduino, along with various technologies such as ZigBee, BLE, and 5G. The authors introduce the DELTA ML model, applied to a multi-layer radiomap, improving vertical and horizontal localization based on fingerprinting. The model initiates the localization process by estimating 2D positions and then determining 3D positions through recursive predictions, promising advances in navigating complex indoor environments. ML is at the forefront also in [38,39], where several deep learning models, including Artificial Neural Network (ANN), LSTM, and CNN, are employed to improve the performance of fingerprint-based localization algorithms over traditional methods. The effectiveness of these systems is demonstrated through experimental validation using both open-source datasets and real-world testing.

*Connectivity Information*—Range-free localization algorithms, particularly those that exploit connectivity information, are known for their low computational and practical complexity, as well as their cost-effectiveness, which makes them attractive for IoT applications. This technique also has low granularity, relying on hop dependence rather than physical distance. Unlike the other techniques in this category, fingerprinting has a granularity that depends on the mapping process during the offline phase, i.e., how many points in the environment are included in the radio map. Therefore, the cost-effectiveness advantages come at the cost of lower accuracy in location estimation than the other approaches discussed in this research [27].

**Figure 4:**
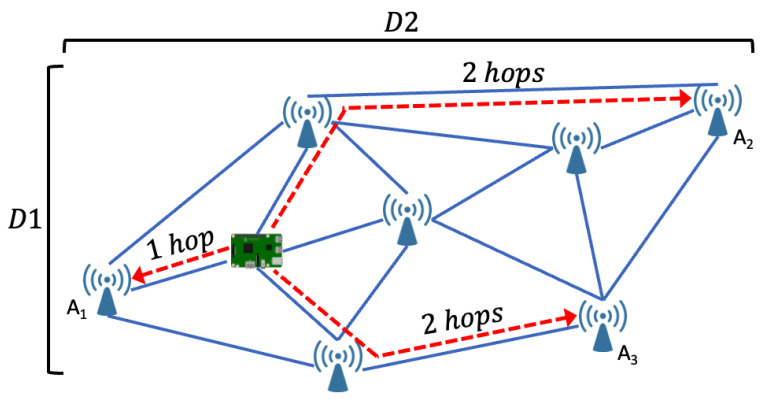
DV–Hop localization topology.

A widely used approach in this category is Distance Vector–Hop (DV–Hop), which employs a hop-based propagation model in which anchor nodes begin the transmission of location information with a hop count set to zero. The nodes update their tables as packets are received, replacing hop counts with lower values. This iterative process continues until all nodes determine the minimum hop count for each node. Next, the anchor nodes estimate the average hop distances and disseminate this information to neighboring nodes. Target nodes then use these data to calculate distances to anchor nodes through a three-way method, refining their position estimates within the network [40]. A representative topology of this approach is shown in Figure 4.

Recent literature presents several approaches aimed at improving traditional DV–Hop algorithms. In [41], a new MATLAB implemented algorithm is proposed, which incorporates distance error correction metrics to improve accuracy and minimize errors in radio range-free localization. Shifting the focus to energy efficiency, [42] introduces a three-step algorithm: the initial discovery of nodes through improved MAC-level communication, the categorization of discovered nodes into direct and indirect sets to minimize communication energy consumption, and finally the introduction of a correction factor to reduce localization errors. Error minimization is the main objective in [43], where four innovative localization algorithms incorporating the DV–Hop algorithm with PSO are presented. Simulations demonstrate the superiority of these approaches over standard ones. In contrast, [44] proposes three new approaches, integrating DV–Hop and Chicken Swarm Optimization (CSO), aiming to establish higher efficiency and accuracy compared to approaches employing PSO. Finally, to improve the accuracy and simplify the complexity of DV–Hop-based algorithms, the authors of [45] propose two algorithms. One is based on centralized connectivity and optimizes accuracy by considering real connectivity constraints. The other, based on distributed connectivity, achieves near-optimal performance in distributed networks by focusing on the real connectivity within two hops, without including the connectivity of all nodes.

### 3.2. Radio Range-Based

**Figure 5:**
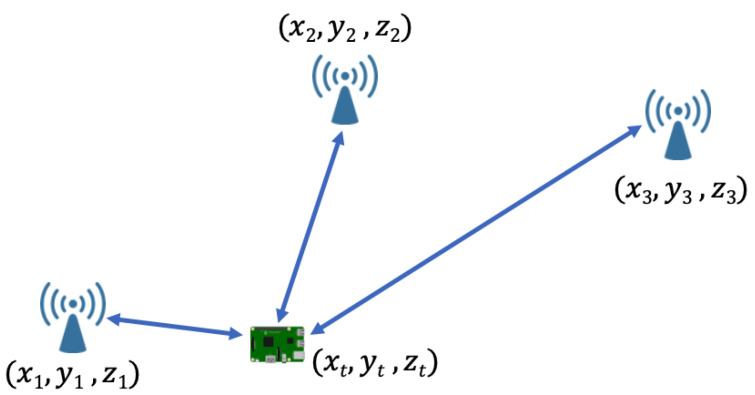
Proximity-based localization.

*Proximity*—This localization technique relies on measurements that indicate whether two devices are within a certain radius or connected, rather than determining precise distances. In fact, the estimated position of the target is given by the position of the nearest connected anchor as illustrated in Figure 5. Due to its simplicity and minimal requirements in terms of energy and computational resources, this technique is widely used in the literature [46]. It is particularly suitable for IoT scenarios where highly accurate location estimates are not a primary concern.

In their study, the authors of [47] explored the potential of Bluetooth Low Energy (BLE) beacons to improve indoor localization by comparing three of the most widely used devices in the literature and evaluating their power consumption and proximity accuracy. The same technology was used in [48], where its effectiveness in improving the user experience inside a museum was demonstrated. Another promising technology for IoT was proposed in [49], where different Sigfox-based proximity localization methods were introduced. The initial approach estimates location by exploiting the known position of the strongest Sigfox base station in reception, while the second and third algorithms introduce the notion of clusters and work on it to improve performance. In [50], the authors evaluated the accuracy of a proximity-based localization algorithm by comparing it with other radio range-based and radio range-free methods. The evaluation was carried out in a large-scale urban environment using a public Narrowband Internet of Things (NB-IoT) network.

**Figure 6:**
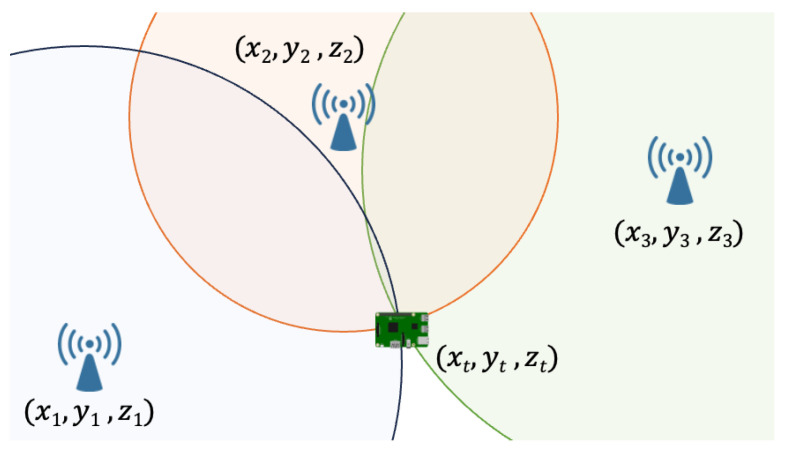
Principle of multilateration-based localization that uses measurements of RSS, ToF, ToA, TDoA, PDoA.

*Received Signal Strength (RSS)*—A common approach in IoT for position estimation is Received Signal Strength (RSS)-based multilateration. As depicted in Figure 6, it involves measuring the RSS from the established anchor nodes, estimating the distances between the target and each anchor, and using multilateration to determine the position of the target. Typically, multilateration approaches use algorithms based on Least Squares (LS), a method in which a system of equations formed by the target–anchor distances is solved by minimizing the sum of the squares of the differences between the actual and estimated values, thereby determining the most probable position. In general, RSS-based approaches offer a cost-effective solution by leveraging wireless transceivers embedded in devices, the existing network architecture, and requiring minimal computational power, aligning with IoT [51] requirements.

For these reasons, methods belonging to this category are widely explored in the literature. In [52], a new approach addresses the challenges posed by measurement imperfections and anchor reliability using Dempster–Shafer theory, non-Gaussian probability density functions, and realistic modeling of RSS deviations. Experimental results show excellent performance in various IoT environments, from residential to laboratory environments. While staying in the indoor environment, in [53], an innovative algorithm is introduced that exploits the principles of multilateration and Non-linear Least Squares (NLS). Experimental tests reveal its superior performance compared with existing algorithms, especially in terms of accuracy. The paper [54] explores RSS-based localization, employing two distinct technologies: Wi-Fi and Long-Term Evolution (LTE). The choice between these technologies is contingent upon the indoor or outdoor nature of the target location. The studies presented in [55,56] analyze the application of Long-Range (LoRa) technology for RSS-based localization. Tested in different scenarios, both outdoor and indoor, these studies demonstrate the promising potential of LoRa technology in terms of position estimation accuracy and robustness to various forms of interference. Exploring intricate and futuristic IoT scenarios, the authors of [57] address the challenges of WSNs in underwater and underground environments. They emphasize the importance of addressing the directionality issues associated with these localization approaches in such scenarios.

*Time of Flight (ToF) and Time of Arrival (ToA)*—Methods based on Time of Flight (ToF) and Time of Arrival (ToA) exploit the signal propagation time to calculate the distance between the anchors and the target, followed by multilateration to estimate the position. This operating principle aligns with the concept illustrated in Figure 6. Although technically attractive, these methods have a significant limitation due to the sensitivity to clock synchronization errors between devices. Practical application is further constrained by the impact of obstacles that deflect the emitted signals, presenting additional challenges in indoor [10] scenarios.

In [58], the authors introduced a ToF algorithm that surpasses traditional approaches. This algorithm integrates joint clock synchronization, LS estimation for emission and arrival time, and Maximum Likelihood Estimation (MLE) using a Gaussian noise model to overcome the challenges associated with this technique. To address some synchronization challenges, a system employing BLE technology for continuous time synchronization nodes was presented in [59]. Experimental tests demonstrated a synchronization error on the order of microseconds, affirming the system’s compatibility with ToF-based positioning. The work in [60] introduced an embedded optimization approach based on nonlinear LS and two-way ToA measurements. Experimental results on a UWB network demonstrated the achievement of subdecimal localization accuracy, making it suitable for applications with high requirements.

*Time Difference of Arrival (TDoA)*—Given the challenges posed by ToA and ToF, in the recent literature, alternative approaches with similar principles are explored, with Time Difference of Arrival (TDoA) emerging among the solutions. This methodology simplifies the implementation by requiring synchronization only between anchor nodes [61]. Distances are now computed by analyzing differences in signal arrival times from different anchors, and then the position is estimated through multilateration in line with the procedure depicted in Figure 6.

Another significant problem inherited from ToA and ToF is the vulnerability concerning Non-Line-Of-Sight (NLOS) paths, causing errors in estimating the true distance between nodes. In [62], this challenge is tackled through two formulations: one jointly estimates the source position and NLOS error, reducing the upper bounds of errors; the other introduces a balancing parameter and transforms the measurement model to overcome issues caused by the triangle inequality in traditional robust LS. The NLOS problem was addressed also in [63], where the authors introduced a new method based on optimization with Semi-Definite Programming (SDP) to mitigate these errors. The authors of [64] addressed another propagation problem that is given by strong multipath channel components in indoor environments. The work introduced a method that exploits broadband signal generation on low-power narrowband transceiver chips. The proposed approach, validated through a measurement campaign with Software-Defined Radio (SDR) platforms, demonstrates effective usability within the bandwidth limits of the 2.4 GHz ISM band while achieving excellent performance. The study in [65] focuses on exploring UWB localization using TDoA in scenarios where anchors are placed very close together and, consequently, the possibility of being placed symmetrically, thus compromising accuracy. This challenge is overcome by a strategy based on selecting subsets of anchors and fusing estimations across multiple subsets. Building upon this, the research is further expanded in [66], where the proposed algorithm is applied to applications associated with mobile target tracking. The study in [67] demonstrates the feasibility of TDoA-based localization with low-power, low-cost technologies such as LoRa, in indoor and outdoor scenarios. The study particularly focuses on the localization of individuals, specifically those belonging to vulnerable groups, making it ideal for applications related to human search and rescue. LoRa is also the focus of [68], where five TDoA algorithms are validated through simulations and field measurements.

*Phase Difference of Arrival (PDoA)*—As mentioned earlier, each signal has arrival time differences based on the distance between the target and the anchor, resulting in phase differences. This provides the basis for the Phase Difference of Arrival (PDoA) method, which determines the distances between the target and the anchor using these differences [69]. Then, the position is estimated through multilateration as shown in Figure 6.

The study detailed in [70] explores a new approach to extend the range of RFID tracking using low-power Tunneling Tags through the PDoA method in the frequency domain. The proposal is shown to be effective in both indoor and outdoor scenarios, demonstrating excellent performance in terms of accuracy, robustness to interference, and power efficiency. In [71], the advantages of employing multi-frequency PDoA in Low-Power Wide-Area Networks (LPWANs) were investigated. The proposed adaptation addresses limitations related to temporal resolution, providing increased accuracy and robustness without compromising energy efficiency and spectrum utilization. The use of a multi-frequency approach was also employed in [72], which presented a new method for indoor autonomous vehicle localization. The developed scheme integrates dual-frequency PDoA, MLE, and a localization algorithm based on SDP and Kalman filtering, achieving excellent performance in terms of accuracy and resilience to interference of any nature. Staying within the context of challenging scenarios, the authors of [73] once again show the potential of UWB technology by proposing a high-precision PDoA positioning method for elderly care in smart homes. The proposed method provides excellent experimental results, requiring minimal NLOS compensation and demonstrating its robustness in these challenging environments.

**Figure 7:**
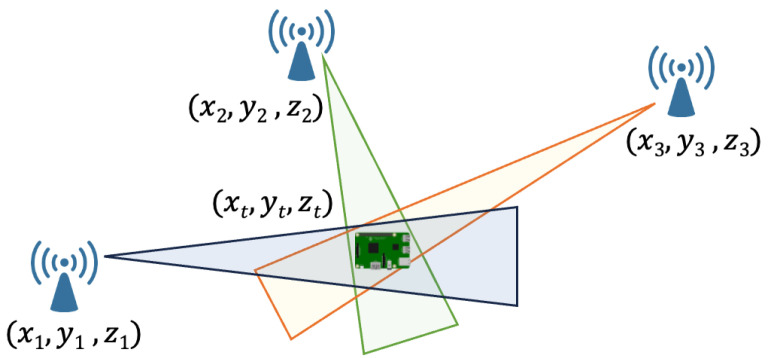
AoA-based localization.

*Angle of Arrival (AoA)*—In Angle of Arrival (AoA) localization, the position is estimated at the center of gravity within the intersection area formed by the sight triangles between the target and the anchors as in Figure 7. The method is based on simple angular geometric considerations but, while offering high accuracy, its effective integration into the IoT environment is limited by the complexities arising from the need for specialized hardware, such as antenna arrays and high signal-processing capabilities [74]. In addition, AoA typically requires an unobstructed Line Of Sight (LOS) and can be sensitive to environmental conditions, potentially limiting its reliability in specific scenarios.

Despite this, there are several works in the literature that work on this promising approach. In [75], the authors introduced a new two-step iterative algorithm for AoA estimation and subsequent refinement through multilateration. The algorithm was tested in a network using BLE and demonstrated excellent results compared to standard approaches. Similarly, [76] focused on BLE technology, presenting an approach based on CNN to address challenges such as noise, multipath effects, and path loss. To address the same interference-related challenges, ref. [77] presented a confidence-aware AoA-based localization system. The proposed work addresses the problem of variable reliability in AoA estimation, which affects the performance of Wi-Fi-based localization, using mathematical approaches and decision weighting based on measurement confidence. Exploring the promising Ultra-WideBand (UWB) technology in this field, ref. [78] presented AnguLoc, an efficient system designed to overcome duplex ambiguity and unknown skew, with the aim to improve accuracy and reduce packet exchange. Similarly, in [79], the authors addressed the challenges of the AoA method by proposing a framework for 5G and IoT networks. This framework integrates NLS curve fitting, and Kalman and Gaussian filtering to effectively mitigate these interferences. The work in [80] shifted the focus from the previously discussed problems. The authors specifically addressed the complexity of integrating this approach into small IoT devices, emphasizing the limitations of current miniaturization strategies. They proposed an innovative solution based on Multiple-Input and Multiple-Output (MIMO) antennas, making this approach more accessible for IoT.

### 3.3. Hybrid Solutions

IoT localization based on hybrid approaches is attracting increasing interest in the literature due to its promising capabilities. Through the skillful combination of different techniques, such as the combination of proximity and multilateration-based approaches, and the integration of data from various technologies such as Wi-Fi and Bluetooth, these approaches improve the accuracy, adaptability, and resilience of location systems.

*Joint Techniques*—Focusing on combining different techniques, a new algorithm integrating Round-Trip Time (RTT) and Wi-Fi RSS measurements was presented in [81], achieving improvements in the accuracy and scalability of the localization system. A hybrid RSS method was also explored in [82], where it was combined with AoA measurements to obtain a 3D localization scheme with high accuracy and robustness, addressing problems related to nonconvexity and computational complexity. Similarly, the combination of RSS and AoA was exploited in [83], presenting an algorithm designed to be effective and scalable, especially in harsh outdoor IoT environments. Focusing on critical parameters typical of these localization systems, the authors of [84] integrated two similar techniques, ToF and TDoA, aiming to combine the accuracy advantages of the former with the energy efficiency of the latter. Following a similar logic, the work [85] proposed a methodology that integrates TDoA and PDoA with PSO, achieving significant improvements in localization performance over conventional methods that rely solely on TDoA.

*Data Fusion*—Shifting the focus to hybrid algorithms that combine different transmission technologies, a recurring trend emerges: the prevalent adoption of Wi-Fi. This technology, due to its widespread integration in most IoT infrastructures, arises as a common element in all significant works in this category. In [86], it was shown how the integration of a Wi-Fi architecture, characterized by shaded regions, with BLE beacons leads to improved indoor location accuracy through data fusion. Similarly, in [87], an LS-based localization algorithm combining Wi-Fi and Bluetooth was introduced, showing the ability of the dual technology to produce more accurate results and a resilient system. In addition, the authors offered an open-source Wi-Fi/Bluetooth dataset, a valuable resource for researchers in the field. To exploit the advantages of the respective technologies, the approaches presented in [88,89] introduced a hybrid strategy that blends a Wi-Fi infrastructure with a strategic deployment of UWB beacons. In both papers, the authors showed how this combination achieves very accurate estimates, effectively overcoming the individual limitations of each technology. Concluding, the study outlined in [90] introduced a hybrid positioning system tailored for diverse seamless location applications, integrating Wi-Fi, Bluetooth, ZigBee, and UWB protocols. Tested in a typical operational environment, the system showcases superior performance across all aspects.

### 3.4. Comparative Analysis of Techniques

The IoT localization literature encompasses a wide range of methodologies, as outlined in this review and summarized in Table 3. Each approach presents a unique set of strengths and limitations, requiring a judicious selection based on the specific use case and its requirements. The suitability of each technique depends on several metrics, most notably accuracy, implementation cost and computational complexity intricately linked to energy consumption, as well as coverage and scalability.

One of the most widely used methods in practical applications is fingerprint localization. This technique exhibits commendable performance across multiple metrics, including *accuracy*, *complexity*, and consequently *energy consumption*, as well as *coverage* and *scalability* [91]. These characteristics make this technique particularly suitable for complex environments, such as Smart Cities [92] and Industrial IoT (IIoT)-related scenarios [93]. However, it is critical to recognize that the *implementation costs* associated with this method can be significant, especially as coverage requirements increase [91]. Belonging to the same category, connectivity information-based methods also show similar characteristics in terms of *implementation costs*, *complexity*, and consequently *energy consumption*, as well as *coverage* and *scalability* [94]. However, they exhibit lower *accuracy* compared to both fingerprint- and distance-based localization approaches, making them unsuitable for application scenarios with stringent accuracy requirements [95].

Moving on to the range-based category, localization using proximity stands out as the simplest one, offering advantages such as *cost-effectiveness*, low *computational complexity* and *energy efficiency*, albeit at the expense of lower *accuracy* [96]. Common applications include those with simple functionality, such as ensuring safety in simple environments [97] or implementing Smart Housing solutions [98]. These applications typically require determining the proximity of the target to specific areas within a given environment, rather than precise localization.

All other range-based approaches reviewed in this survey, such as RSS, AoA, ToA, etc., offer high levels of *accuracy*, *coverage*, and *scalability* [21]. However, they have the disadvantage of high *implementation costs*, *computational complexity*, and *energy consumption* [99]. The application of these localization techniques mirrors that of fingerprint-based technology, but achieves superior results in dynamic scenarios, demonstrating increased adaptability and effectiveness.

Finally, hybrid approaches, which are gaining interest in the research community, show versatility and can be adapted to different domains depending on the specific requirements of the application. These approaches skillfully highlight and address the inherent strengths and weaknesses of the techniques and technologies used.

Table 4 summarizes the discussion in this section by listing the key performance metrics for each technique analyzed in this study. Regarding the values of the performance metrics in Table 4 (i.e., low/medium/high), the performed analysis is not in terms of absolute values, but it is a comparison between approaches. As an illustrative example, if complexity and energy consumption are considered metrics, connectivity information techniques have medium performance and can be assumed as a benchmark. Therefore, fingerprinting, which is labeled as low/medium, has similar but slightly lower complexity and consumption. Multilateration techniques, instead, which are labeled as high, are characterized by higher complexity and consumption with respect to techniques exploiting connectivity information. Similar considerations hold for the other metrics. This supplement provides valuable information about these technologies and helps determine the best approach for specific applications and requirements.

## 4. Threats Models, Detection and Mitigation

Numerous attacks represent a threat to IoT localization systems, operating at various stages of the process. This review focuses on attacks that specifically affect the physical layer of the architecture as outlined in Figure 1. These attacks, with different targets, can lead to the disruption of location service (*availability*) or incorrect location due to tampering actions (*authenticity*). This section examines the primary threat models for each localization approach and presents the most promising solutions proposed in the literature to address them. For summary purposes, Table 5 is provided at the conclusion of this section.

### 4.1. Availability

The attacks against availability are orchestrated to disable the system from being able to determine the position of the target. Denial-of-service (DoS) approaches belong to this category, and the jamming attack is the one most frequently observed in the literature [100]. Jamming represents a form of DoS attack that obstructs the channel, preventing other nodes from using it to communicate. Among the various localization techniques described in this study, no one is immune to this particular type of attack [101].

Let us consider the RSS-based localization technique as an example. The target–anchor distance estimation is based on the RSS measured from the target and involves calculations based on the signal propagation model [102]. In the case of a jamming attack, the Signal-to-Noise Ratio (SNR) of the receiver decreases significantly. Consequently, the target unaware of the attack overestimates the distance from the anchors, potentially causing substantial errors or, in the worst case, making position estimation no longer affordable.

Numerous papers in the literature focus on the challenge of jamming attacks in the broad context of IoT and WSNs, offering various approaches to detect and mitigate them [102,103,104,105,106,107]. Turning our attention to the more specific domain of IoT localization systems, in [108], the authors introduce AS-DILOC, a consensus-based iterative distributed algorithm featuring an abandonment strategy to mitigate packet loss in communication links during DoS attacks. This ensures accurate sensor localization, regardless of the attacker’s strategy.

### 4.2. Authenticity

The main security concern for IoT localization systems involves authenticity. Cybersecurity threats targeting this essential aspect focus on gaining control of one or more anchors within the scenario or infiltrating the network by assuming a benevolent facade. Once inside the system, attackers try to compromise the accurate distance estimation by the compromised anchors. This is achieved by providing manipulated information, such as tampered reference positions, or by manipulating transmission parameters, such as transmission power [109].

Within this survey, we have classified attacks against authenticity into four primary categories based on their execution methods: Spoofing, Sybil, Byzantine, and Wormhole attacks.

*Spoofing*—In a Spoofing attack, a malicious node adopts the identity of a benign anchor node, typically duplicating its MAC address. This deceptive scheme allows the malicious node to impact the localization process in a variety of ways. Several papers in the literature propose solutions for this type of attack. The paper [110] introduced SecureLoc, a prototyping platform specifically designed to evaluate secure location methods in indoor environments. The research includes an in-depth analysis and evaluation of Spoofing attacks, illustrating the effectiveness of the platform in evaluating security measures. The paper [111] addressed the problem of Spoofing in the context of fingerprint-based localization. The authors presented BERT-ADLOC, a system designed to detect fake fingerprints during database updates and defeat attacks during online inference. The scheme was tested on a BLE fingerprint-based system, showing excellent localization performance against adversaries in both phases.

*Sybil*—The Sybil attack, particularly prevalent in IoT location systems that rely on connection information, is a highly destructive threat. The main goal of these attackers is to create multiple false identities, thereby causing a misleading perception of numerous nodes within the network, resulting in the devastation of the perceived topology [112]. Once again, the scientific community has proposed several solutions to address the problem. The authors in [113] addressed Sybil attacks that target connectivity-based localization by introducing a secure version of DV–Hop. This adaptation enables the detection and mitigation of these threats while preserving estimation accuracy even under attack. In the paper [114], the authors introduced PrSLoc, a new algorithm designed to improve robustness against Sybil attacks in RSS-based localization systems. The algorithm achieves this by incorporating Approximate Point-In-Triangulation and Differential Privacy mechanisms to safeguard the identity of nodes.

*Byzantine*—A Byzantine attack is characterized by the intrusion of an attacker taking control of one or more nodes within the network to disrupt the proper functioning of the localization process. These malicious nodes engage in activities such as providing false information or tampering with the transmission parameters, thus compromising the accuracy of position estimates [115]. There are many promising solutions in the literature to counter this highly aggressive form of attack. The article [116] proposed four techniques: Weighted Least Square (WLS), secure WLS (SWLS), and L1-based techniques, namely LN-1 and LN-1E. Demonstrating significant advantages in uncoordinated attacks, WLS and SWLS detect and mitigate malicious nodes, while LN-1E prevents disruptions in coordinated attacks by treating the location problem as a plan adaptation problem. A WLS-based algorithm was proposed also in [117], showing to be particularly effective in countering the impact of attackers who manipulate the transmission power of anchors. In the work [118], these threats were formulated as an intractable maximum a posteriori problem, considering a practical model of attack and uncertainties. The proposed algorithm iteratively approximates the true posterior distribution, providing closed-form estimates of position and velocity while identifying malicious nodes. The paper [119] introduced a robust two-step feature selector, employing an AP trust model and Manifold Learning to ensure resilience against Byzantine attacks.

*Wormhole*—In a Wormhole attack, the adversary strategically places two malicious nodes in the network, establishing a dedicated low-latency channel between them. This channel facilitates deceptive communication, causing nodes within range of one malicious node to inaccurately perceive the proximity of the other node as well, as if they were only one hop away [120]. In DV–Hop-based localization, this attack causes significant damage, leading the scientific community in this area to analyze and propose mitigation solutions. In [121], a new secure DV–Hop algorithm was introduced. By delegating data message transmission to neighbor nodes and using a trust-based strategy, the algorithm significantly improves attack detection rates, reduces localization errors, and minimizes energy costs as evidenced by experimental results. Through the integration of centralized localization, the identification of malicious nodes employing a Single-Class Support Vector Machine, and localization recovery, the authors of [122] introduced a Secure Optimized Localization algorithm adept at countering Wormhole attacks. The authors in [123] enhanced resilience against Wormhole attacks by introducing an algorithm founded on the principles of Farkas’ lemma. This approach enables the identification and mitigation of Wormhole with higher accuracy compared to several existing methods in the literature.

*Generalized Solutions*—There are also some works in the literature that present solutions aimed at solving multiple types of attacks among those mentioned above. For instance, in [124], a secure localization algorithm leveraging blockchain was outlined. This algorithm aims to safeguard the precision of declared anchor locations and the authenticity of exchanged data, effectively mitigating the impact of diverse types of attacks. Similarly, in [125], the authors presented a blockchain-based fingerprint localization scheme that establishes a tamper-proof real-time database of electromagnetic fingerprints. Through simulations, they demonstrated the feasibility and robustness of the scheme against Spoofing and Sybil attacks. Staying in the domain of fingerprint-based localization, the paper [126] introduced SE-Loc. This technique, based on Semi-Supervised Learning, provides robustness to various types of attacks through continuous learning of scenario characteristics. Similar logic was also used in [127] to detect routing-type threats, such as Wormhole and Sybil, using a hybrid ML approach optimized for distance, location, and data communication. The same attacks were also discussed in [128], where innovative detection algorithms based on the concept of the highest-rank common ancestor were introduced and validated.

**Table 5:** Summary of works proposing solution-specific threats divided by category.

Threat Model	Year	Reference	Proposed Solutions in the Field of		
			Radio Range-Based	Fingerprinting	Connectivity Info.
DoS	2021	[108]	X	X	X
	2023	[126]		X	
Spoofing	2019	[110]	X		
		[124]	X	X	X
	2021	[111]		X	
	2023	[126]		X	
		[125]		X	
Sybil	2019	[124]	X	X	X
	2020	[113]			X
		[128]			X
	2023	[114]		X	
		[126]			X
		[127]		X	
		[125]	X		
Byzantine	2019	[124]	X	X	X
	2021	[116]	X		
		[118]	X		
		[119]	X	X	
		[117]	X		
	2023	[126]		X	
Wormhole	2019	[124]	X	X	X
	2020	[128]			X
	2022	[121]			X
	2023	[122]			X
		[123]			X
		[126]		X	
		[127]			X

## 5. Summary and Guidelines

In line with the objectives outlined in the introduction of the survey, this section offers insights and personal conclusions on the current state of the literature. Key findings of the manuscript are listed below and then discussed in detail:

- A substantial majority of articles employ radio range-based methods.
- There is growing interest in hybrid approaches that jointly exploit different techniques and use the fusion of data from different technologies to improve localization performance.
- Radio range-based techniques typically adopt a dual approach involving both experimental tests and simulations.
- Researchers are increasingly focused on security aspects. Works addressing these issues address specific threats related to particular localization techniques.
- Range-based approaches are susceptible to Byzantine attacks, connectivity information-based methods are vulnerable to Wormhole and Sybil attacks, and fingerprinting encounters a variety of challenges.

Going into detail and focusing on the literature related to IoT localization techniques, significant insights emerge for researchers in this area. First, in quantitative terms, it is evident that a *substantial majority of the articles employ radio range-based methods* compared to radio range-free methods as shown in Table 3. This prevalence underscores the inherent advantages associated with these techniques, including easier implementation, higher accuracy, suitability, and adaptability to diverse and dynamic environments. One notable trend emerging from this survey is the *increasing prevalence of hybrid approaches*. Researchers are increasingly proposing solutions that join different techniques, such as combining radio range-based algorithms with fingerprinting, and various technologies, such as leveraging Wi-Fi and Bluetooth through data fusion. Analyzing this trend, we expect the literature on hybrid methods to grow in the coming years. As this survey has shown, the combination of different techniques and technologies promises significant improvements, not only in terms of accuracy but also in terms of robustness. In particular, focusing on hybrid approaches using data fusion, we believe that future research should focus on exploring the use of multiple short-range technologies. These technologies could be used in combination or as backups for each other to improve the robustness of the systems. Furthermore, incorporating both short-range and long-range technologies can improve the scalability of the systems and make them more flexible to different situations.

**Figure 8:**
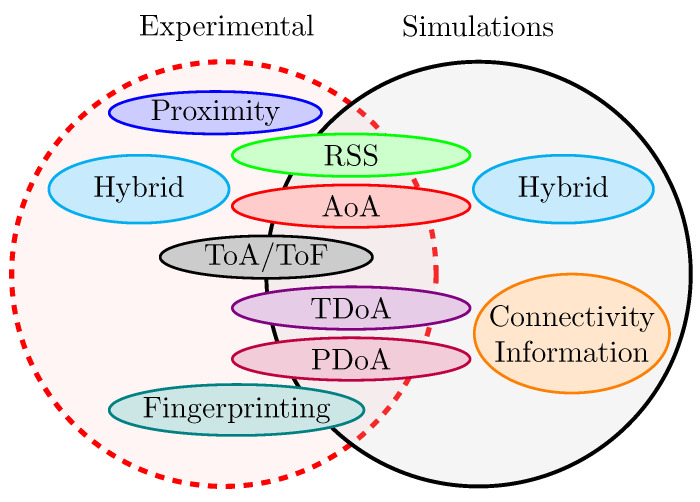
Distribution of papers according to the methodologies used.

As part of the review, special attention was paid to a crucial aspect in the evaluation of literature references: the methodology used to validate the works. The question was whether the studies were based on real tests, simulations, or both at different stages of development. The importance of this aspect lies in the significant influence that the chosen methodology has on the reproducibility of the research results. By clarifying these characteristics, we offer valuable support to researchers, helping them identify work that may be valuable in the development of their proposals. The Venn diagram presented in Figure 8 provides valuable insights into the methodologies employed in different papers, offering significant statistical insights that outline key trends within the literature. From a statistical point of view, a prevalent trend can be seen. The papers employing radio range-based techniques *predominantly adopt a dual approach involving both experimental tests and simulations*. This is a consequence of the typical development flow of radio-based localization algorithms, which involves an initial simulation phase to validate the theoretical framework before moving on to field experiments. These involve considerable time and cost, as well as introducing practical challenges due to the use of IoT technologies such as Wi-Fi, BLE, UWB, LoRa, and others, in harsh environments. For this reason, in the literature, field experiments are limited to cases where there is a high level of confidence that the algorithm works as expected. In contrast, in the domain of radio range-free localization techniques, particularly those that rely on connectivity information, there is a clear bias toward the use of network simulators. This bias is inherent in these techniques, which rely on network data rather than physical measurements to estimate target location.

**Figure 9:**
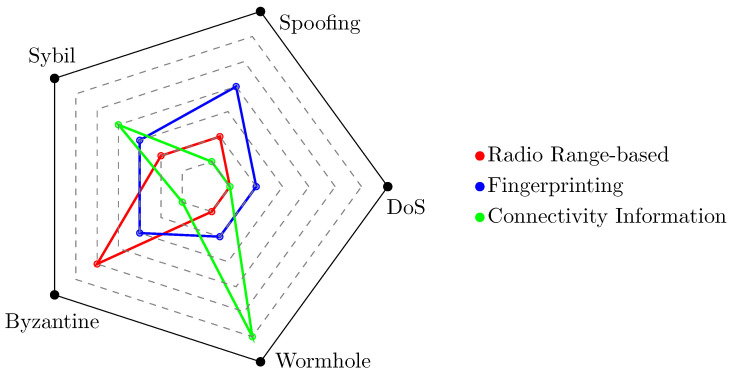
Distribution of the impact of major attacks on localization techniques.

Moving on to reviewing the literature related to threat models, detection, and solutions, several interesting statistics can be extracted, and diverse conclusions can be drawn. First, as illustrated in Table 5, given the number of papers in the recent literature, one can observe an *increasing sensitivity of researchers regarding security aspects*. Furthermore, one can see that the proposed solutions are typically *specialized in addressing a specific type of attack for a particular localization technique*. By delving deeper into individual localization techniques, our analysis provides valuable insights into the potential threats associated with each. This information reported in our survey is particularly important for researchers who specialize in a specific technique, as it enables them to be aware of potential risks and easily identify solutions documented in the literature. The radar diagram shown in Figure 9 serves as a comprehensive visual representation of the various threats associated with IoT location techniques. Each vertex of the polygon corresponds to a specific threat, and the proximity of the data points to these vertices designates the increased vulnerability attributed to these attack methods according to the reviewed literature. Note that each vertex of the inner polygons quantitatively represents a single work analyzed in the literature. From this analysis, it can be concluded that radio range-based techniques exhibit heightened *susceptibility to Byzantine attacks*, making them critically vulnerable to unauthorized access and anchor control. Fingerprint-based techniques, as reported in the literature, face a *variety of challenges*, underscoring the need for robust security measures that account for all potential risks. Connectivity-based techniques, by their nature, are primarily *vulnerable to Wormhole and Sybil attacks*. Consequently, an expanding body of literature is dedicated to addressing and mitigating this specific vulnerability.

## 6. Conclusions and Future Directions

In this paper, we conducted a comprehensive study of localization techniques in the IoT domain, with a focus on identifying and addressing their main vulnerabilities. Our survey first presented the various types of localization methods found in the literature, offering insights into the advantages, disadvantages, and typical methodologies associated with each category. Unlike previous survey approaches, our work was intended to be a comprehensive resource for researchers, combining the technical details of implementing the various techniques with insights into potential security challenges and their solutions. Accordingly, we examined threat models and corresponding identification and mitigation strategies, filling a gap in the literature that traditionally separates the two.

The general lesson that emerged from our survey underscores the growing interest of academia in LBS, which consequently extends to IoT localization. In the dynamic IoT landscape, characterized by devices that require less and less human interaction, the importance of context awareness emerges as a critical factor. As a result, the literature proposes a high number of papers with increasingly accurate and robust localization techniques. This trend has been particularly pronounced in recent years, with more than 100% growth observed between 2020 and 2024 compared to the previous decade as shown by statistics obtained using the methodology described in Section 2.1. As the interest in a particular technology grows, researchers are faced with the challenge of ensuring the security, reliability, and accuracy of the systems involved. As a consequence, the scientific community is dedicated to providing a variety of solutions to the numerous attacks that have occurred in recent years. These solutions range from integrating safeguards or countermeasures into localization technologies to creating hybrid algorithms that increase the system’s robustness. In conclusion, through this comprehensive investigation of IoT localization, we have attempted to chart a course by highlighting the current progress, potential challenges, and areas that require further investigation.

Looking ahead, as seen with the emergence of artificial intelligence and ML, other promising technologies such as blockchain [129] and quantum computing [130] will further improve the performance and robustness of IoT localization systems. These advances promise not only to increase system performance but also to enable new LBS and applications previously thought to be infeasible.

## Figures and Tables

**Table 1:** List of used acronyms (in alphabetical order).

Acronym	Definition	Acronym	Definition
ANN	Artificial Neural Network	AoA	Angle of Arrival
AP	Access Points	BLE	Bluetooth Low Energy
CNN	Convolutional Neural Network	CSI	Channel State Information
CSO	Chicken Swarm Optimization	DoS	Denial-of-Service
DV–Hop	Distance Vector–Hop	IIoT	Industrial Internet of Things
IoT	Internet of Things	LBS	Location-Based Services
LoRa	Long Range	LOS	Line Of Sight
LPWAN	Low-Power Wide-Area Network	LS	Least Squares
LSTM	Long Short-Term Memory	LTE	Long-Term Evolution
M2H	Machine-to-Humans	M2M	Machine-to-Machine
MIMO	Multiple-Input and Multiple-Output	ML	Machine Learning
MLE	Maximum Likelihood Estimation	NB-IoT	Narrowband Internet of Things
NLOS	Non-Line Of Sight	NLS	Non-linear Least Squares
PSO	Particle Swarm Optimization	RIS	Reconfigurable Intelligent Surfaces
RTT	Round-Trip Time	RSS	Received Signal Strength
SDP	Semi-Definite Programming	SDR	Software-Defined Radio
SNR	Signal-to-Noise Ratio	SWLS	Secure Weighted Least Squares
TDoA	Time Difference of Arrival	ToA	Time of Arrival
ToF	Time of Flight	UWB	Ultra-WideBand
WLS	Weighted Least Squares	WSN	Wireless Sensor Network

**Table 3:** Summary of work categorized by the IoT localization technique.

Topic	Year	Reference	Type of Work
Proximity	2018	[47,49]	Experimental
	2020	[48,50]	
RSS	2018	[52]	
	2019	[55]	
		[56]	Simulation/Experimental
	2022	[53]	
		[57]	Simulation
	2023	[54]	Experimental
AoA	2019	[75]	Simulation/Experimental
		[77]	Experimental
	2020	[78,79]	Simulation/Experimental
	2021	[76]	Experimental
	2022	[80]	Simulation
ToA/ToF	2019	[58,60]	Simulation/Experimental
	2021	[59]	Experimental
TDoA	2019	[62]	Simulation/Experimental
	2020	[67]	Simulation
		[68]	Simulation/Experimental
	2021	[63]	Simulation
		[65]	Simulation/Experimental
	2022	[64]	Experimental
	2023	[66]	Simulation/Experimental
PDoA	2019	[70]	Experimental
	2020	[73]	
	2021	[71]	Simulation
	2022	[72]	Simulation/Experimental
Fingerprinting	2019	[34]	Experimental
	2020	[37]	Simulation/Experimental
		[38]	Experimental
	2022	[35,36,39]	
Connectivity Information	2019	[41,42]	Simulation
	2020	[45]	
	2021	[43]	
	2023	[44]	
Joint Techniques	2019	[81]	Experimental
		[82,84]	Simulation
	2020	[85]	
	2021	[83]	Experimental
Data Fusion	2019	[86,88]	
	2020	[90]	
	2022	[89]	
	2023	[87]	

**Table 4:** Comparison of localization techniques.

Technique	Accuracy	Implementation Cost	Complexity and Energy Consumption	Coverage and Scalability
Fingerprinting	Medium/High	Medium/High	Low/Medium	High
Connectivity Information	Low	Medium/High	Medium	High
Proximity	Low	Low	Low	Low/Medium
Multilateration-based	Medium/High	High	High	Medium/High
